# CCR9 Is a Key Regulator of Early Phases of Allergic Airway Inflammation

**DOI:** 10.1155/2016/3635809

**Published:** 2016-10-04

**Authors:** C. López-Pacheco, G. Soldevila, G. Du Pont, R. Hernández-Pando, E. A. García-Zepeda

**Affiliations:** ^1^CBRL, Ciudad de México, Mexico; ^2^Departamento de Inmunología, Instituto de Investigaciones Biomédicas, Universidad Nacional Autónoma de México, 04510 Ciudad de México, Mexico; ^3^Departamento de Patología Experimental, Instituto Nacional de Ciencias Médicas y Nutrición “Salvador Zubirán”, Tlalpan, 14080 Ciudad de México, Mexico

## Abstract

Airway inflammation is the most common hallmark of allergic asthma. Chemokine receptors involved in leukocyte recruitment are closely related to the pathology in asthma. CCR9 has been described as a homeostatic and inflammatory chemokine receptor, but its role and that of its ligand CCL25 during lung inflammation remain unknown. To investigate the role of CCR9 as a modulator of airway inflammation, we established an OVA-induced allergic inflammation model in CCR9-deficient mice. Here, we report the expression of CCR9 and CCL25 as early as 6 hours post-OVA challenge in eosinophils and T-lymphocytes. Moreover, in challenged CCR9-deficient mice, cell recruitment was impaired at peribronchial and perivenular levels. OVA-administration in CCR9-deficient mice leads to a less inflammatory cell recruitment, which modifies the expression of IL-10, CCL11, and CCL25 at 24 hours after OVA challenge. In contrast, the secretion of IL-4 and IL-5 was not affected in CCR9-deficient mice compared to WT mice. These results demonstrate for the first time that CCR9 and CCL25 expressions are induced in the early stages of airway inflammation and they have an important role modulating eosinophils and lymphocytes recruitment at the first stages of inflammatory process, suggesting that they might be a potential target to regulate inflammation in asthma.

## 1. Introduction

Allergic asthma is a chronic disease that affects more than 300 million people worldwide [1]. Its prevalence and mortality have been more common in the recent decades and it became an important health issue due to its increasing medical care expenses and a reduction in worker productivity. It is estimated that there will be more than 100 million of new asthmatics in the next decade [2–5].

Allergen-triggered airway inflammation mediated by specific IgE is the most common feature of asthma [2]. Airway inflammation is mediated by the recruitment of granulocytes and Th2 lymphocytes [6]; both are described as the main cellular effectors of the inflammatory process and are regulated by Th2 cells-derived cytokines that are expressed by epithelial and inflammatory cells [7].

The complexity of cell trafficking during lung inflammation is tightly regulated by chemokines [8]. In this context, it has been established that overexpression of certain chemokine receptors is correlated with the localization and activation of inflammatory cells during and after an allergen challenge. Increasing evidence has supported the role of chemokine receptors in allergic airway inflammation; however, the involvement of CCR9 in asthma remains unclear.

CCR9 and its unique ligand, CCL25 (thymus-expressed chemokine, TECK), were originally described in thymus where they were shown to play a role in thymocyte development [9–11]. Also, their homeostatic expression in small intestine is related with cell homing to the gut. Moreover, the involvement of CCR9 under inflammatory conditions has been widely described in the gut [10, 12]. However, its expression, function, and regulation in the lungs are unclear.

The expression of CCR9 in inflammatory cells that are recruited to the lungs has been described. After* in vitro* stimulation with proinflammatory mediators, human eosinophils-derived cell lines upregulate the expression of CCR9 and respond to CCL25 in chemotaxis assays [13]. Also, CCR9 is upregulated in peripheral blood eosinophils of asthmatic subjects [14]. Moreover, inflammatory macrophages which are crucial cells in allergic inflammation upregulate CCR9 expression in the inflammatory microenvironment [15]. Dendritic cells are some of the most important effectors in early stages of airway sensitization. In these cells, CCR9 expression is upregulated by IL-4 [16]; however, the effects of other Th2-derived cytokines in this induction and its impact on the regulation of the inflammatory process have not been characterized.

We have previously shown that a subpopulation of CD4+ CD25+ FoxP3+ T-lymphocytes have regulatory functions and depend on CCR9 expression to control pathogen-induced inflammation in the gut [17]. Therefore, the aim of this work was to analyse the role of CCR9 in regulating cell recruitment and modulating the inflammatory process during airway sensitization. Consistent with previous studies, we report that CCR9 is expressed by eosinophils and T-lymphocytes. In addition, we demonstrate that CCR9-deficient mice (KO) sensitized with OVA showed an impaired recruitment of granulocytes during the first stages of inflammation resulting in a diminished inflammatory response. These data suggest that CCR9 expression plays a key role in the modulation of cell recruitment into the airways during pulmonary inflammation in asthma.

## 2. Materials and Methods

### 2.1. Animals

Six- to eight-week-old C57BL/6 female mice were used in all experiments and maintained under pathogen-free conditions. Water and food were supplied* ad libitum*. CCR9-deficient mice were kindly provided by Dr. M. A. Wurbel (Boston Children's Hospital, Boston, MA, USA). All experimental procedures involving animals were handled in strict accordance with good animal practices as defined and approved by the Animal Experimental Bio-Ethics Guidelines of the Instituto de Investigaciones Biomédicas, Universidad Nacional Autónoma de México.

### 2.2. Allergic Airway Inflammation Murine Model

To induce airway inflammation we followed a previously reported protocol with minor modifications [18, 19]. Briefly, mice were treated by intraperitoneal administration with 10 *μ*g of ovalbumin (OVA, Sigma Chemical, MO, USA), adsorbed to 10 mg of alum (Pierce, NJ, USA), and suspended in saline solution (SS) on days 0 and 8. On days 15 to 20, mice received aerosolized OVA 1% (USB, Ohio, USA) during 30 minutes. On day 34, mice were again nebulized with OVA 5%. Mice were euthanized 6, 24, and 48 hours later when indicated. Mice treated only with SS were used as controls.

### 2.3. Measurement of OVA-Specific Mouse IgE by ELISA

For detection of serum OVA-specific IgE, 96-well ELISA plates (Santa Cruz, TX, USA) were coated with 20 *μ*g of OVA in 0.1 M carbonate buffer (pH 8.3). Plates were blocked one hour with PBS-SFB 2%. Samples were diluted 10-fold and then added to the wells. Antibodies were detected with rat anti-mouse IgE specific antibody (Zymed, CA, USA), followed by the addition of a secondary anti-rat biotinylated antibody (BioLegend, CA, USA). Avidin-HRP (BD OptEIA, USA) was added and reaction was developed with tetramethylbenzidine substrate (KPL, MD, USA) and stopped by adding H_2_SO_4_ 2 N. Optical density of samples was measured at 450 nm (OD450) using a Modulus II microplate reader (Promega, WI, USA).

### 2.4. Bronchoalveolar Lavage Fluid (BALF) Cell Analysis

BALF obtained from challenged mice was collected at 2, 6, 24, and 48 h after the last OVA administration as indicated. Briefly, 800 *μ*L of PBS/EDTA 0.05 M solution was repeatedly introduced into the mice lungs and collected until recovered volume was 4 mL. BALF was centrifuged at 1500 rpm for 10 min at 4°C. Cells were suspended in 1 mL of PBS and total number of cells were quantified using standard staining protocols and differential cell types were determined on cytospun cells stained with Diff Quick (Dade Behring Inc., Newark, DE) and then examined by microscopy. Morphological analysis of cells distinguished lymphocytes, macrophages, eosinophils, and neutrophils. Remaining cells were processed for flow cytometry analysis.

### 2.5. Cytokines Determination in BALF

Levels of IL-4, IL-5, TNF-*α*, and IL-10 cytokines were measured in BAL supernatants using commercial ELISA kits (ELISA Max™, BioLegend, San Diego, CA, USA) according to the manufacturer's instructions. Additionally, a murine MultiAnalyte ELISArray kit (Qiagen Inc., Valencia, CA) was used to quantitate the levels of 12 proinflammatory cytokines and performed according to the manufacturer's instructions.

### 2.6. Flow Cytometry Analysis

To analyse chemokine receptor expression by FACS, cells were incubated first with blocking solution (mouse serum in PBS) during 20 minutes, followed by a second incubation with a biotinylated mouse anti-CCR9 antibody (1 *μ*g/10^6^ cells) (eBioscience, CA, USA) in FACS buffer (PBS containing 2% fetal bovine serum and 0.1% NaN_3_) for 30 min at 4°C. Finally cells were incubated with a solution of streptavidin-APC or streptavidin-PerCP during 30 min at 4°C in the dark.

For phenotypic detection of other cell populations, single cell suspensions were preincubated with a mixture of anti-CD16/CD32 antibodies (BioLegend, CA, USA) during 10 min at room temperature. Following first incubation and several washes, cells were incubated with either anti-CD4-PeCy7, anti-CD8-PE, anti-Gr1-PeCy5 (BioLegend, CA, USA), or anti-Siglec (BD, CA, USA) antibodies in FACS buffer for 30 min at 4°C (according to manufacturer's suggestions). Finally, samples were analyzed in Attune® Acoustic flow cytometer (Life Technologies, Thermo Fisher Scientific, MA, USA). Analysis of data was performed with FlowJo 8.7 software (Tree Star, Inc.).

### 2.7. Histological Analysis

Mice were euthanized, and the lungs fixed by transcardiac and intratracheal instillation with 4% paraformaldehyde. After fixation, lungs were embedded in paraffin and processed for histochemical analysis. Lung sections (7 *μ*M) were stained with haematoxylin and eosin (HE) and periodic acid-Schiff (PAS). For morphometric analysis, slides were examined in a Leika microscope (Leica Microsystems Inc., Buffalo Grove, IL, USA). Images were taken from 5 different fields from four animal samples and analyzed using Leika microscope imaging software (Leica Microsystems).

### 2.8. Immunohistological Analysis of Chemokine Expression

Briefly, paraformaldehyde fixed lung tissues were embedded in paraffin and processed for immunohistochemical analysis. Sections were deparaffinized and rehydrated with ethanol gradients. Antigen retrieval was performed by microwave oven heating in a 0.01 M sodium citrate solution; endogenous peroxidases were inactivated with H_2_O_2_ and blocked with universal buffer (Biogenex, CA, USA). Purified goat anti-mouse CCL25 antibody (R&D) and control goat IgG isotype (R&D), were used as recommended by manufacturer. Sections were incubated overnight; after several washes, secondary peroxidase-labeled anti-goat IgG was added during 1 hour at room temperature. Finally, sections were 5 times washed with PBS/BSA 0.05% and revealed with DAB (Vector Laboratories, CA, USA).

### 2.9. Real-Time PCR

Total RNA from tissues was purified with Trizol (Invitrogen, CA, USA). cDNA synthesis was performed using 2 *μ*g of total RNA as a template, M-MLV (Promega, Madison, WI, USA), and OligodT (Invitrogen) according to manufacturer's instructions. Real-time PCR was performed using the Power SYBR® Green PCR Master Mix (Applied Biosystems). Primer sequences used for amplifications were as follows: CCR9: F: 5′-TCCGAAGGGATCTGGTGAAG-3′, CCR9: R: 5′-GAATGAAACCCACTGGGC C-3′; CCL25: F: 5′-CCAAGGTGCCTTTGAAGACT-3′, CCL25: R: 5′-TCCTCCAGCTGGT GGTTACT-3′; CCL11: F: 5′-TCCACAGCGCTTCTATTCCTG-3′, CCL11: R: 5′-G GAGCC TGGGTGAGCCA-3′; CCR3: F: 5′-TTCTCACCAGGAAGAAACGGA-3′, CCR3: R: 5′-GG AGGTGACTGAGGTGATTGC-3′; IL-10: F: 5′-TTTGAATTCCCTGGGTGAGAA-3′, IL-10: R: 5′-GGAGAAATCGATGACAGCGC-3′; IL-5: F: 5′-ACCTTGGCACTGCTTTCTACTC AT-3′, IL-5: R: 5′-AGAAACTCTTGCAGGTAGTCTAGG-3′ and *β*-actin as an internal control gene F: 5′-GGGTCAGAAGGATTCCTATG-3′, R: 5′-GGTCTCAAACATGATCTGGG-3′. Gene expression analysis was performed relative to *β*-actin and calculated by using the 2^−ΔΔCt^ method [20].

### 2.10. Data Analysis

Significant differences between groups were evaluated using one-way analysis of variance (ANOVA), followed by Dunnett's multiple comparisons test using GraphPad Prism version 6.0 (GraphPad Software, La Jolla, California, USA). The values are reported as mean ± SD. Differences with a *p* value equal to or below 0.05 were considered as statistically significant.

## 3. Results

### 3.1. Characterization of the Inflammatory Cells Induced by OVA-Stimulation in CCR9-Deficient Mice

**Figure 1:**
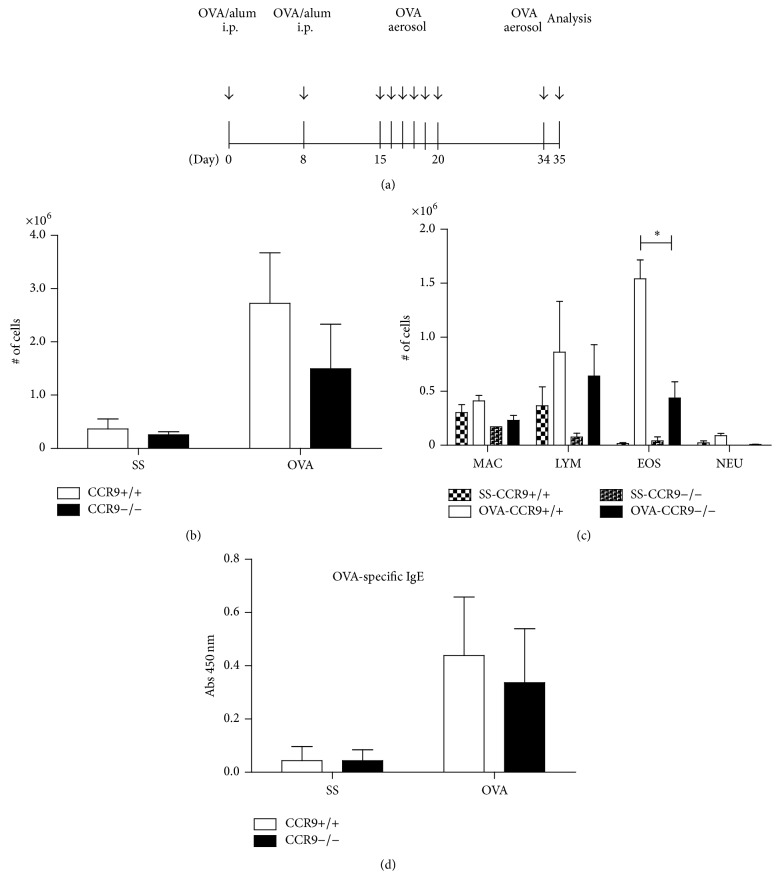
Leukocyte recruitment is impaired in BALF in absence of CCR9. (a)* Wild type* (WT) and* knockout* (KO) mice were first sensitized with OVA/alum intraperitoneally. Control animals received saline solution (SS). OVA was aerosolized as indicated. Mice were sacrificed 24 h after the last antigen challenge. (b) Total number of cells from BALF WT and KO mice was quantified. (c) Collected BALF cells were stained with Wright's stain and lymphocytes (LYM), macrophages (MAC), eosinophils (EOS), and neutrophils (NEU) were distinguished morphologically. (d) OVA-specific IgE was determined by ELISA in serum samples from mice. Data represent mean ± SD and are representative of 4 independent experiments. *n* = 3-4 for each group (^*∗*^ *p* < 0.05 when compared with WT group).

Our first approach to analyse the regulatory role of CCR9 in allergic lung inflammation* in vivo* was to use a murine model of ovalbumin-induced airway inflammation (Figure 1(a)). Wild type (WT) and CCR9-deficient (KO) mice were sensitized and challenged with OVA. The inflammatory response was induced and analyzed 24 hours after the last OVA-challenge. The absence of CCR9 resulted in impaired cellular recruitment, as observed by a diminished cell count in bronchoalveolar fluid (BALF) (Figure 1(b)). In contrast to SS-treated mice, OVA-challenged mice showed an increase in lymphocytes and eosinophils as reported earlier [19]. Interestingly, there was a reduction (30%) in the total number of recruited eosinophils in BALF of KO mice compared to WT mice (Figure 1(c)). However, we found no differences in OVA-specific IgE production in the sera of CCR9 KO mice compared to WT (Figure 1(d)). Altogether, these results indicate that CCR9 regulates eosinophilic infiltration in the lung in response to OVA.

### 3.2. Reduced Leukocyte Infiltration in the Absence of CCR9

**Figure 2:**
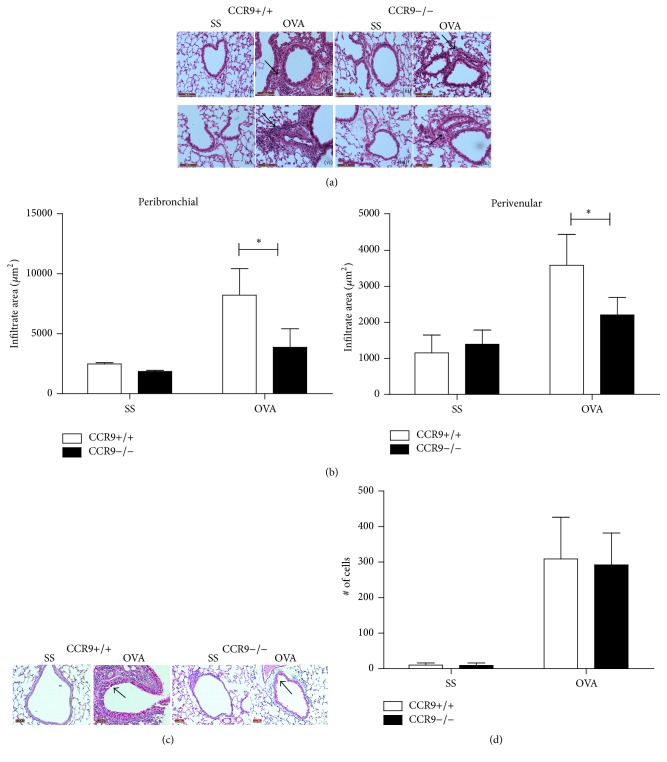
Allergic airway inflammation in the absence of CCR9. Lungs from SS- and OVA-treated mice were fixed and stained with H&E. (a) Representative images from peribronchial (i–iv) and perivenular (v–viii) inflammation. Arrows indicate leukocyte infiltration (magnification 200x). (b) Peribronchial and perivenular infiltration was measured from 5 different fields from each mouse and represented as area of infiltrate. (c) Representative images from WT mice SS-treated (i) and OVA-challenged (ii) and CCR9-KO mice SS (iii) and OVA-challenged (iv). Arrows indicate PAS-positive goblet cells (magnification 200x). (d) Quantification of PAS-positive cells from 5 different fields. Images and mean data are representative of 4 independent experiments, *n* = 3-4 for each group (^*∗*^ *p* < 0.05 when compared with WT mice).

Next, we determined the histological features of airway inflammation in CCR9 KO mice. Consistent with the number of inflammatory cells in BALF, we found an increase of leukocyte cell infiltration at peribronchial and perivascular levels in OVA-treated WT and CCR9 KO mice, primarily mononuclear cells and eosinophils (Figure 2(a)). Morphometrical analysis showed significantly reduced peribronchial infiltration (nearly by 50%) and slight decrease at the perivenular level in the absence of CCR9 compared to WT mice. In addition, it has been suggested that chemokines may participate directly in regulating mucin expression. Thus, to examine another feature of the lung inflammatory response, we compared mucus production in WT with CCR9 KO mice by PAS-staining (Figure 2(c)). Our analysis showed that there was no significant difference in the number of PAS-positive goblet cells in each group of mice (Figure 2(d)). These results might indicate that CCR9 has a role in regulating airway infiltration of inflammatory cells but apparently not in regulating mucin expression.

### 3.3. Analysis of BAL Cytokines in OVA-Challenged CCR9-Deficient Mice

**Figure 3:**
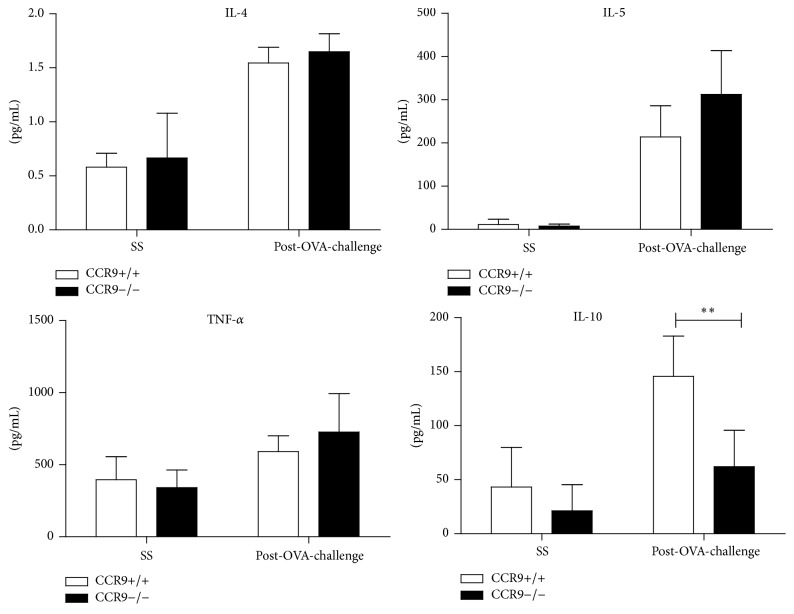
Absence of CCR9 decreases IL-10 production in BALF. BALF cytokines were quantified by ELISA. Levels of IL-4, Il-5, TNF-*α*, and IL-10 represented as mean ± SD. *n* = 4 (^*∗∗*^ *p* < 0.01 when compared with WT mice or when compared each time with control group).

Th2 cytokines are the main regulators of leukocyte recruitment during allergic inflammation. IL-4 is involved in induction of airway hyperresponsiveness and production of specific IgE and T cell-derived IL-5 is one of the key regulators of eosinophilia in lungs. We analysed the levels of IL-4 and IL-5 in the airways of OVA-sensitized CCR9 KO or WT mice and found no significant differences in the levels of either cytokine between CCR9 KO and WT littermates (Figure 3). Moreover, despite the fact that TNF-*α* has been reported to contribute to the recruitment of neutrophils, eosinophils, and lymphocytes in allergic models, TNF-*α* production was not affected in CCR9 KO mice (Figure 3). On the other hand, a strong correlation has been reported between CCR9 expression and a regulatory process in the gut; therefore, the reduction of eosinophil infiltration observed in CCR9 KO mice (Figure 1(c)) might also be associated with cytokine-mediated immunoregulatory events. Interestingly, we found that IL-10 is significantly reduced (more than 60%) in CCR9 KO compared to WT mice (Figure 3). These results indicate that, unlike previous data reported in the gut mucosa, where the absence of CCR9 results in increased inflammation [17], during an allergic inflammatory process in the lung, CCR9 expression correlates with increased leukocyte recruitment, despite the reduced levels of IL-10. Additionally, we analysed the expression of cytokines that have been associated with regulation of inflammation in the lung. Interestingly TGF-beta and IL-17 increased their concentration at 6 h and up to 24 h after OVA-stimulation in the CCR9 KO mice compared to WT (Supplementary Figure 1 in Supplementary Material available online at http://dx.doi.org/10.1155/2016/3635809).

### 3.4. CCR9 Regulates CCL25 Expression after OVA-Stimulation

**Figure 4:**
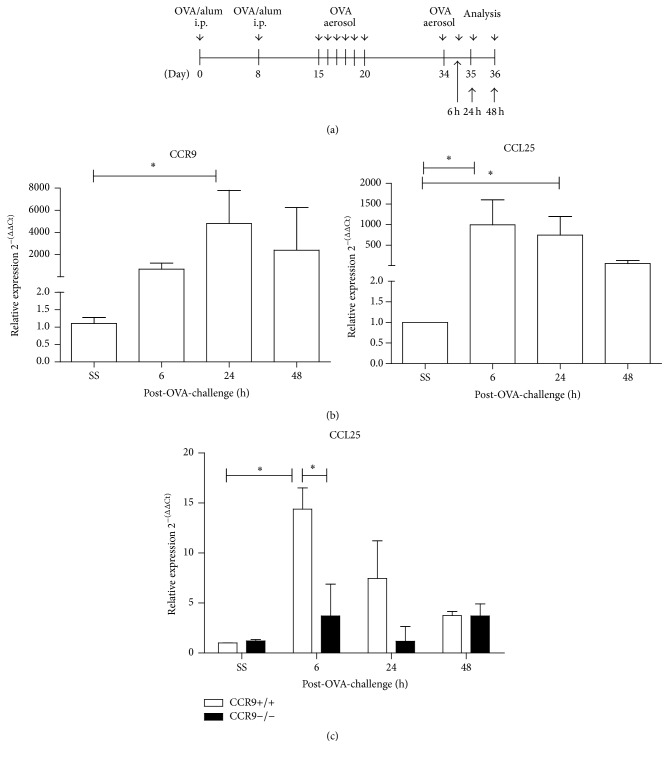
CCL25 expression in the lungs of allergen sensitized mice. (a) Mice were sacrificed 6, 24, and 48 h, respectively, after the last OVA-challenge. (b) CCR9 and CCL25 expression in lungs were quantified by real-time RT-PCR. (c) CCL25 expression in lungs of CCR9 KO and WT mice. Data are representative of 3 independent experiments and represent mean ± SD. *n* = 2-3 for each group (^*∗*^ *p* < 0.05 when compared with WT mice or when compared each time with control group).

Analysis of leukocyte infiltration after 24 hours after the final OVA-treatment suggested that eosinophils and inflammatory mediators evaluated are dependent on CCR9 expression. A detailed study on the kinetics of the CCR9/CCL25 axis expression during lung inflammation has not yet been performed. Therefore, we next analysed the kinetics CCR9 expression using a modified model of airway sensitization (Figure 4(a)). Very low levels of CCR9 mRNA expression were found in lung tissue by real-time RT-PCR, although they were detectable at 6 h after the last OVA-challenge reaching the maximum level of expression at 24 h after OVA-challenge (Figure 4(b)). In contrast, we found that CCL25 expression was upregulated very early after allergen challenge (6 h) and was starting to decrease at 24 h and further downregulated at 48 h. To investigate if CCL25 expression might be regulated by CCR9, we analyzed expression of the CCL25 in CCR9 KO and WT mice, before and after OVA-challenge. Interestingly, we detected very low levels of CCL25, in OVA-treated KO mice compared to WT (Figure 4(c)), suggesting that CCR9 expression is important for CCL25 induction in the airways during allergic inflammation. We also have analysed CCL25 expression by IHC in our lung inflammation model after 0, 6, and 24 OVA-stimulation (Figure S2). As demonstrated in the qRT-PCR experiments, there was an increase in the expression of CCL25 in the WT mice as early as 6 h after OVA-challenge; in contrast, there is a reduction in CCL25 expression in the CCR9−/− mice at all times tested.

### 3.5. CCR9 Modulates the Expression of Other Mediators of Lung Inflammation

**Figure 5:**
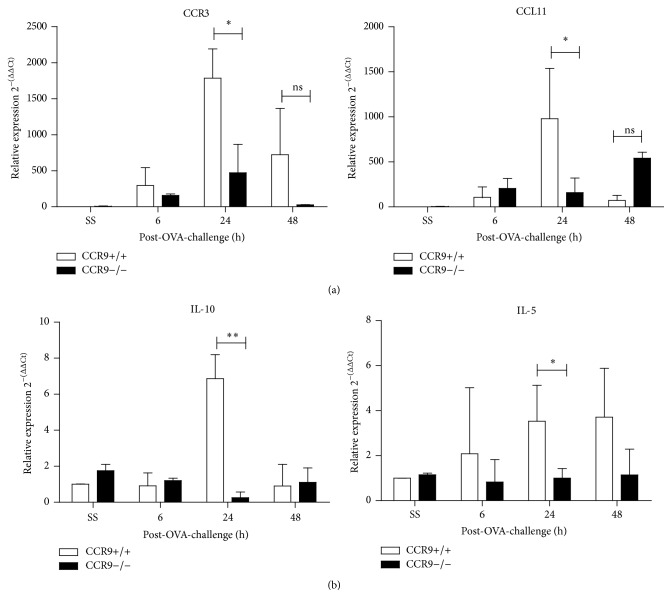
Gene expression of lung chemokine receptors and cytokines during OVA-induced inflammation in absence of CCR9. Expression of CCR3, CCL11, IL-5, and IL-10 in WT and CCR9−/− mice was analyzed at 6, 24, and 48 h after OVA-stimulation. Data are representative of 3 independent experiments and represent mean ± SD. *n* = 2-3 for each group (^*∗*^ *p* < 0.05, ^*∗∗*^ *p* < 0.01 when compared with WT mice or when compared each time with control group).

Our results suggested that CCR9 might regulate the expression of other inflammatory mediators in the lung. Thus, we analysed the expression of CCR3 and one of its cognate ligands, CCL11, known to be important attractors of eosinophils and Th2 lymphocytes during allergic inflammation in the lungs. As shown in Figure 5(a), in WT mice, there was an increase in the mRNA levels of CCR3 and CCL11 since the first 6 h after the last OVA administration and significant decrease after 48 h (Figure 5(a)). However, comparison between WT and KO revealed that both molecules were severely reduced in absence of CCR9 (Figure 5(b)), suggesting an indirect effect of CCR9 in eosinophils and T-lymphocytes recruitment.

Next, we tested two related cytokines: IL-5, which is involved in activation, recruitment, and apoptosis-resistance of eosinophils, and IL-10 that is an immunoregulatory cytokine. As shown in Figure 5(a), mRNA levels of both cytokines are increased at 24 h after OVA-stimulation in WT mice. Surprisingly, both mRNA levels of IL-5 and IL-10 were either reduced or abrogated at all times tested after OVA-stimulation in the absence of CCR9 when compared with WT littermates (Figure 5(b)). Thus, these data indicate that there is important dependence on CCR9 in the expression of molecules that are important for the establishment of an allergic airway inflammation characterized by the recruitment of eosinophils and T-lymphocytes.

### 3.6. Eosinophil Recruitment to the Lung Is Diminished in the Absence of CCR9

**Figure 6:**
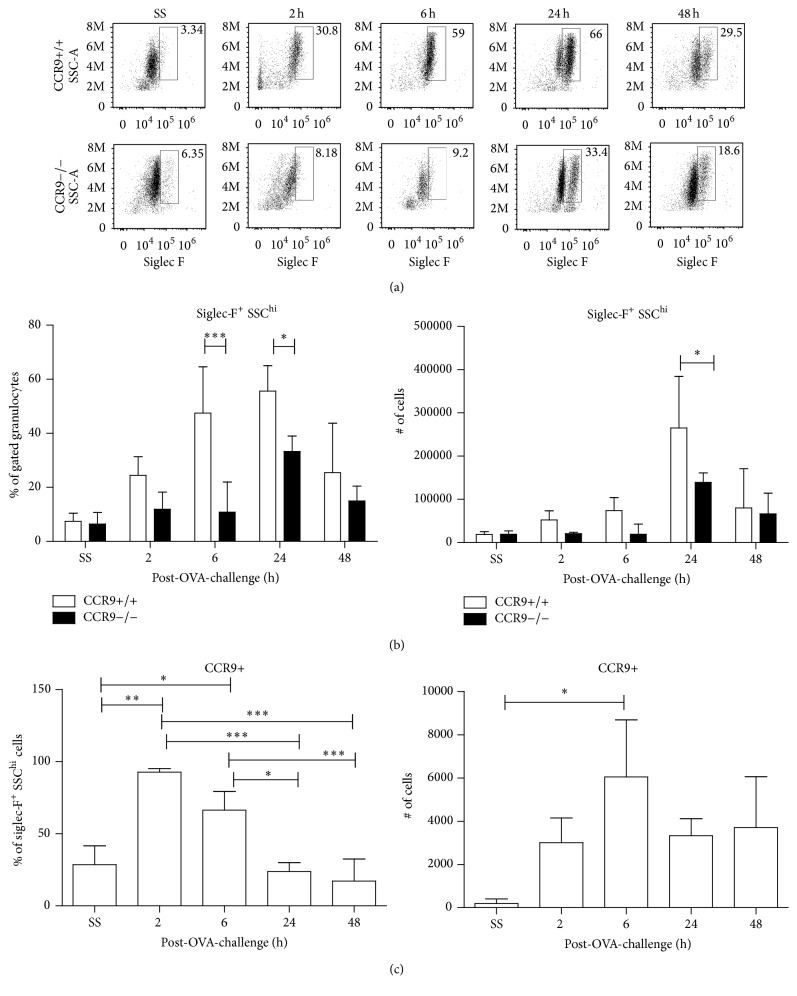
CCR9 expression in eosinophils regulates their recruitment to inflamed lung. 2, 6, 24, and 48 h after the last OVA boost, BALF from OVA-sensitized/challenged mice was collected and analyzed by FACS. (a) Siglec-F^+^ SSC^hi^ cells were plotted. (b) Quantitation of eosinophils in WT and KO mice during inflammation. (c) Percentage and CCR9-expressing eosinophils in WT mice. Data are representative of 2 independent experiments and represent mean ± SD. *n* = 3–5 for each group (^*∗*^ *p* < 0.05,  ^*∗∗*^ *p* < 0.01, and ^*∗∗∗*^ *p* < 0.001 when compared with WT mice or when compared each time with control group).

As CCR3 and CCL11 expression were regulated by CCR9 and a reduction in eosinophil recruitment to the lungs in the BALF of CCR9 KO mice was determined (Figures 1(b) and 1(c)), we further analysed the kinetics of eosinophils recruitment (Gr1+, Siglec-F+ cells) in the lungs of CCR9 KO or WT mice after aerosolized OVA by FACS. Data showed that although eosinophils started to increase at 6 h in both groups of mice, at 24 h after OVA-treatment, there was a significant reduction in CCR9 KO compared to WT mice, both in percentage and in total numbers (Figures 6(a) and 6(b)) which correlates with our previous findings by morphological characterization in BALF (Figure 1(c)) (65% versus 40%).

The mechanisms involved in the regulation of CCR9 expression in eosinophils are unclear. It has been reported that, under* in vitro* activation conditions, CCR9 and other chemokine receptors are upregulated. To obtain a better understanding about a role for CCR9 in eosinophils migration into the BALF after OVA-challenge, we analyzed CCR9 expression in these cells by flow cytometry. Our data showed that there is an increase in the population of CCR9+ Siglec-F+ eosinophils only 6 h after OVA-stimulation. However, we found no statistical differences in Siglec-F expression in the absence of CCR9 after OVA-challenge (Figure S3), which coexpress CCR3 during lung recruitment. Chemokine receptor expression is severely reduced as control littermates at 24 h during inflammation (Figure 6(c)), which suggests that CCR9 is importantly expressed under inflammatory stimulation. Since recruitment of eosinophils to the airways is tightly regulated by chemokine receptors such as CCR3 and CCR5 along with the expression of inflammatory mediators such as CCL5, CCL11, CCL22, and CCL24 chemokines and IL-5, our findings that the CCR9/CCL25 axis may also participate in eosinophils recruitment at the very early hours after OVA-stimulation might contribute to a better understanding of the complex immunoregulation of airways inflammation.

### 3.7. T-Lymphocytes Recruitment into the Lungs Is Affected in the Absence of CCR9

It has been well established that Th2-lymphocytes are involved in IL-5 secretion in the inflamed lung after allergenic stimulation [18]. As previously mentioned, IL-5 and Th2-like cytokines are the major modulators of allergic inflammation, inducing the recruitment and activation of eosinophils in the airways. Thus, we next investigated whether the absence of CCR9 may also result in impaired T-lymphocyte recruitment to the lungs. To achieve this, we assessed lymphocyte subpopulations in BALF of CCR9 KO and WT littermates by flow cytometry.

**Figure 7:**
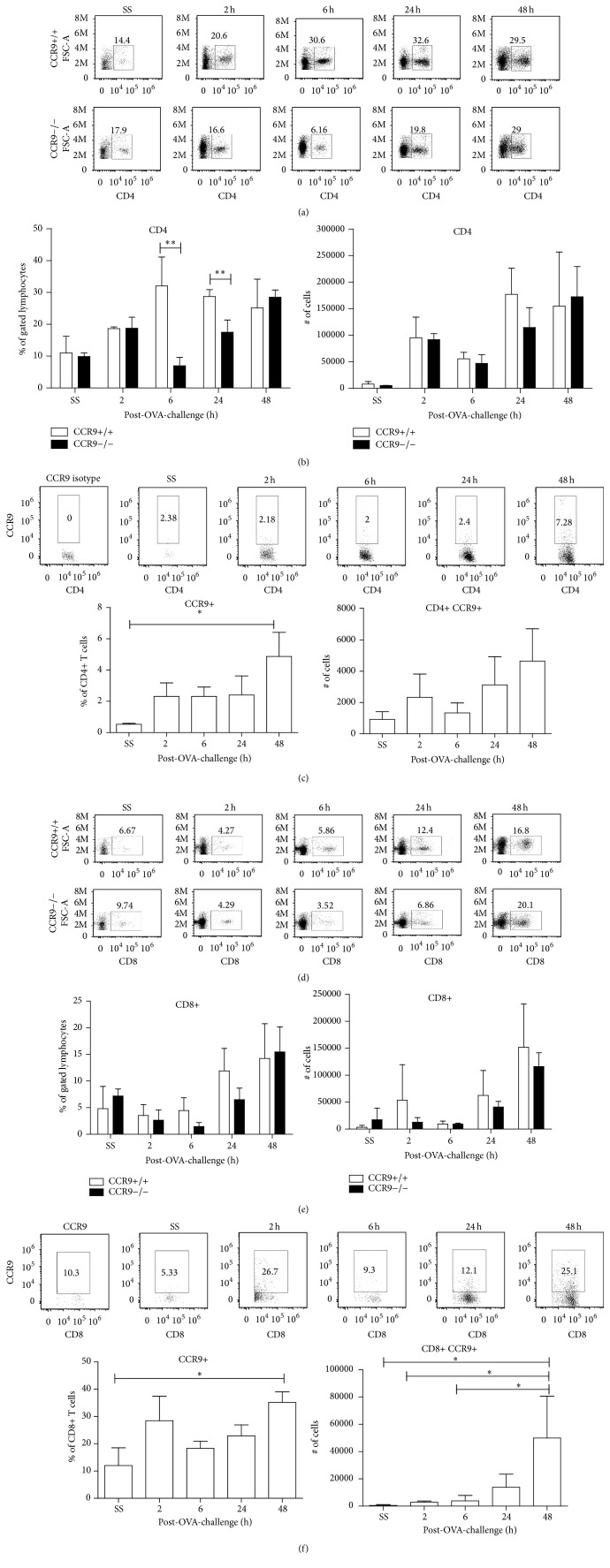
Lymphocyte recruitment during airway inflammation is affected in absence of CCR9. 2, 6, 24, and 48 h after the last OVA-challenge, BALF from sensitized mice was collected and FACS analysis of (a) CD4 and (d) CD8 T-lymphocytes is shown. Percentage of CD4+ (b) and total number (e) of CD4+ T-lymphocytes gated in region of lymphocytes. CCR9+ cells were analyzed in (c) CD4+ or (f) CD8+ T-lymphocytes. Data are representative of 2 independent experiments and represent mean ± SD. *n* = 3–5 for each group (^*∗*^ *p* < 0.05,  ^*∗∗*^ *p* < 0.01, and ^*∗∗∗*^ *p* < 0.001 when compared with WT mice or when compared each time with control group).

As shown in Figure 7, while the percentage of CD4+ T cells in BALF of WT mice increased after OVA-stimulation, CD4+ T cells in CCR9 KO mice were significantly reduced at 6 and 24 h after OVA-stimulation (Figures 7(a) and 7(b)). Recruitment of CD4+ T cells into the lungs of WT mice correlated with the presence of CCR9+ cells since this subpopulation significantly increased 48 h after OVA-stimulation (Figure 7(c)).

The role of CD8+ T cells in allergic inflammation has also been studied and has been shown to be responsible for the secretion of IFN-*γ* and IL-4 [20]. As shown in Figures 7(d) and 7(e), recruitment of CD8+ T cells in CCR9 KO mice was reduced at 6 and 24 h, but not at 48 h after OVA-stimulation. Recruitment of CD8+ CCR9+ T cells into the lungs increased at very early hours after last OVA-stimulation and remained up to 48 h (Figure 7(f)). Altogether these results suggest that expression of CCR9 is important for the recruitment of CD4+ T and possibly CD8+ T cells during allergen-induced airway inflammation.

## 4. Discussion

Airway inflammation is one of the most important features of allergic asthma [21]. The regulation of allergic airway inflammation is a very complex process. Chemokines and their receptors have been widely associated with allergic process, including asthma. Several CC and CXC chemokines have been associated with the asthmatic phenotype: CCL1, CCL2, CCL3, CCL5, CCL11, CCL24, CCL26, CXCL8, and CXCL10 [22–25]. However the dynamics and kinetics of this regulation are not fully understood in the context of asthma development. Also, chemokine receptors like CCR3, CCR4, and CCR8 have been found to be elevated in asthma patients and experimental murine models [26–32]. CCR3 has been widely studied in both clinical and experimental models of asthma [33]. It has been well established that this receptor contributes to regulation of the immune response in asthmatic airways by recruiting and activating eosinophils, T-lymphocytes, and mast cells [34]. The chemokine receptor CCR4 has also been studied in allergic airway inflammation using murine animal models; although its leukocyte expression might not be crucial for allergic airway inflammation development, it has been associated with recruitment of regulatory T cells to the lung [35, 36]. However, little information exists regarding the role of CCR9 in regulation of lung inflammation. CCR9 is a CC-chemokine receptor that has an important but not essential participation in regulating thymocytes trafficking during lymphocyte development in the thymus [11, 37, 38]. Also, CCR9 is associated with homing of CD4 [16], CD8 [39], *γδ* T cells [40], dendritic cells [41], and B cells [42] to the intestinal lamina propria. Likewise, CCR9 and CCL25 participation in inflammatory diseases has been widely studied. Its expression is upregulated in inflammatory bowel disease and colitis [43–45]. Interactions between CCR9 and its ligand are important in regulating development of small intestine inflammation [46, 47], mainly by attracting gut inflammatory cells. CCR9 also has a role in regulation of apoptosis in tumor cell lines [48] and in cancer patients [49, 50], which makes this receptor a potential chemotherapeutic target in chronic inflammatory diseases.

In this report, we were interested in analysing the role of CCR9 in regulating the inflammatory process during allergic airway inflammation. To achieve this, we analysed a previously reported murine model of allergic airway inflammation. Interestingly, our data showed that leukocyte recruitment to allergic airways was impaired in absence of CCR9, in particular affecting recruitment of eosinophils. Histological analysis demonstrated that both perivascular and peribronchial inflammation were attenuated in CCR9 KO mice, suggesting that CCR9-CCL25 interaction might be important, but not crucial, in activating classical features of airways inflammation.* In vivo* expression of CCR9 in eosinophils is not clear [14] and* in vitro* data has suggested that CCR9 could be induced under an inflammatory stimulus [51]. Another important feature of allergic airway sensitization is an increase in the expression of lung mucins. We analysed the number of PAS-positive goblet cells in the lungs of OVA-stimulated/challenged mice and showed no statistical differences between KO and WT mice. Although few studies have been reported regarding a direct relation between chemokine stimulation and mucin production, it is not clear whether CCL25 may be directly involved in mucin production [52, 53]. Further studies regarding the chemokine-mediated induction of the different types of mucin should be performed, in particular analysing those chemokines that are related to a Th2-type inflammation phenotype such as CCL5 and CCL11. Next, we proceed to investigate the potential role of the CCR9-CCL25 axis in regulating some of the events of the inflammatory allergic process in association with other cytokines. It is clear now that Th2-type cytokines are crucial for allergic development and remodelling [54, 55]. IL-4, IL-5, IL-9, and IL-13 cytokines have a clear effect on target cells such as B cells and eosinophils. In addition, other cytokines such as IL-1, IL-22, IL-33, and TSLP-1 may also activate both Th2 and ILC2 innate cells increasing the complexity of the inflammation process. However, a Th2-type environment alone does not seem to explain the broad spectrum of this disease, in part, because severe asthma is not exclusively associated with Th2 cytokines production [56].

We found that the expression of IL-4 and IL-5 was not modified in the BALF of OVA-stimulated CCR9 KO mice, although there was a significant increase of IFN-*γ* (Supplementary Figure 1) after 6 h after OVA-stimulation, suggesting that Th2 cytokines might not be related to CCR9-mediated signalling and that other mechanisms of regulation are involved in their expression under absence of CCR9.

In contrast, the levels of IL-10 in the BALF of OVA-sensitized mice were reduced in the absence of CCR9 compared to WT. IL-10 is a potent immunoregulatory cytokine produced mainly by a subset of CD4+ T-lymphocytes (Tregs), B lymphocytes, and macrophages [57, 58]. In asthma, there are a number of studies both in patients and in animal models that demonstrate a potential role of IL-10 regulating inflammation in the airways [59], where the main source is CD4+ CD25+ T-lymphocytes. However, in our model no differences were found in the numbers of CD25+ FoxP3+ T cells, indicating that other CCR9-dependent IL-10 producing cells might be involved in the regulation of the inflammatory process in the airways. In this context a dendritic cell-like subpopulation (CD11+c, F480+ MHC Class II+) was diminished in the lung of CCR9 KO at very early time after stimulation (data not shown).

IL-17 and TGF-beta were also modified in the absence of CCR9 (Figure 3 and Supplementary Figure 1). It has been reported that Th2 and Th17 pathways might be regulated in asthma. An increase in IL-17 expression is related to neutrophilic inflammation that is also associated with an increase in the levels of certain CXC chemokines and mucin hyperplasia [60, 61]. In our model, the absence of CCR9 led to a downregulation of the IL-17 levels in the allergic airways although with no significant modification of the levels of IL-4 and IL-5.

CCR9 has been identified in secretory plasma B cells in the gut [41]. We were interested in analysing the levels of IgE, IgG1, and IgG2a serum antibodies (Figure 1, data not shown) and found no significant differences between wild type and* knockout* mice. This is not surprising since the levels of IL-4 in the* knockout* mice were not significantly modified. As it is known, IL-4 is associated with proliferation and differentiation of activated B cells, IgE switching, induction of eosinophil transmigration across endothelium, regulation of Th2 responses in allergic diseases, and effects on the synthesis of chemokines such as CCL11, CCL24, CCL26, and CCL13 [62–64]. Although a correlation between IL-4 and IgE has been proven to be relevant in allergic airway inflammation, in our model, the absence of CCR9 has no impact on the expression of these proteins.

Early phases of allergic airway inflammation (first 4–8 h after allergen stimulation) are characterized by the expression of specific chemokines, cytokines, and growth factors that correlate with the initial infiltration of neutrophils and eosinophils and the activation of mast cells. This recruitment is associated with development of airway hyperresponsiveness (AHR). Experimental models have shown that there is a coordinated regulation between chemokine expression and differential leukocyte recruitment. IL-4, IL-5, IL-13, and TNF-*α* cytokines are upregulated during this phase, playing a key role in AHR development, leukocyte recruitment, and late phase establishment of inflammation, which could persist for weeks [65, 66]. In addition, CC (CCL2, CCL3, and CCL5) and CXC (CXCL1, CXCL2, CXCL5, and CXCL10) chemokines are expressed at early events of airway inflammation (0–8 h after allergen challenge) while CCL8, CCL11, and CCL24 are expressed at later events (from 24 h up to 7 days) [67–69]. Therefore, here we investigated whether the CCR9/CCL25 axis might be involved in the modulation of the kinetics of cell recruitment into the airways during early stages of inflammation after allergen stimulation. Our data showed an upregulation of both CCR9 and CCL25, both at mRNA and at protein levels, as early as 6 h and up to 24 h after allergen challenge. This expression correlated with an increase of lymphocytes and eosinophils. Thus, CCR9/CCL25 expression may contribute to leukocyte recruitment under a proinflammatory microenvironment at very early stages of allergen stimulation in a similar fashion to CCL11. Since CCL25 is constitutively expressed by thymic and small intestinal epithelial cells [70], it is likely that the main source of CCL25 in the lung is also epithelial cells, although it may not be regulated in the same way as other chemokines [71].

In our model, we analysed 2 and 6 h post-OVA-stimulation as key points of early inflammation onset. In the absence of CCR9, an impaired eosinophil recruitment to the lungs was detected. Eosinophilic response postactivation has been widely described both* in vivo* and* in vitro*. This activation results in production of proinflammatory mediators and chemokines; thus a defect in the eosinophil recruitment could be related not only to CCR9 expression but to migratory dependence and activation of eosinophils mediated by CCL25. This process may be crucial for inflammation regulation and resolution. Furthermore, our results show a decrease in the number of CCR9+ Siglec-F+ eosinophils after the first 6 hr. after the OVA-challenge. These cells return to basal levels at 48 hours after stimulation, suggesting that these CCR9+ cells might be specifically important at very early phases of lung inflammation.

Similarly, an allergic airway inflammatory late response (from 1-2 days) is characterized by migration of T-lymphocytes, macrophages, and a second wave of granulocytes. This leukocyte recruitment is regulated by specific cytokines and chemokines that also contribute to further airway remodelling [65, 72]. In our model, absence of CCR9 severely impaired migration into the airways of both CD4+ and CD8+ T cells after 24 hours of OVA-challenge. The total numbers of these cells were similar to that of OVA-stimulated WT controls at 48 hours after last OVA-challenge. It is not clear if this reduction in the number of T cells at early stages of airway inflammation correlates with a regulatory T-lymphocyte phenotype (CD4+ CD25+ FoxP3+) as we have demonstrated earlier in a model of pathogen-mediated inflammation in the colon [17].

Taken together our data indicated that CCR9 regulates allergic airway inflammation by promoting early airway eosinophilia affecting CD4 and CD8 T-lymphocytes recruitment and suggest that this receptor could be considered as an additional therapeutic target in allergic airway inflammation.

## Supplementary Material

Supplementary Figure 1. IL-2, IL-6, IL-12, IL-13, IL-17A, IL-23, IFN-g and TGF-b were determined from BAL by ELISA multiarray. CCR9 deficiency modifies the inflammatory cytokine production specially at 6 hours post OVA challenge.
Supplementary Figure 2. CCL25 expression was analyzed in lung tissue by immunohistochemistry at 6 an 24 hours after challenge. Images demonstrate that CCL25 peaks is at 6 hours and is dependent on CCR9 expression.
Supplementary Figure 3. Phenotypic analysis of eosinophils was performed by FACS in BAL-derived cells. CCR9 expression was determined and Siglec was not altered in the absence of CCR9.
