# Supplementary figures and images for: CCR9 Is a Key Regulator of Early Phases of Allergic Airway Inflammation
Source: Mediators Inflamm. 2016 Oct 4;2016:3635809. doi: 10.1155/2016/3635809 (PMC5067335; doi:10.1155/2016/3635809)

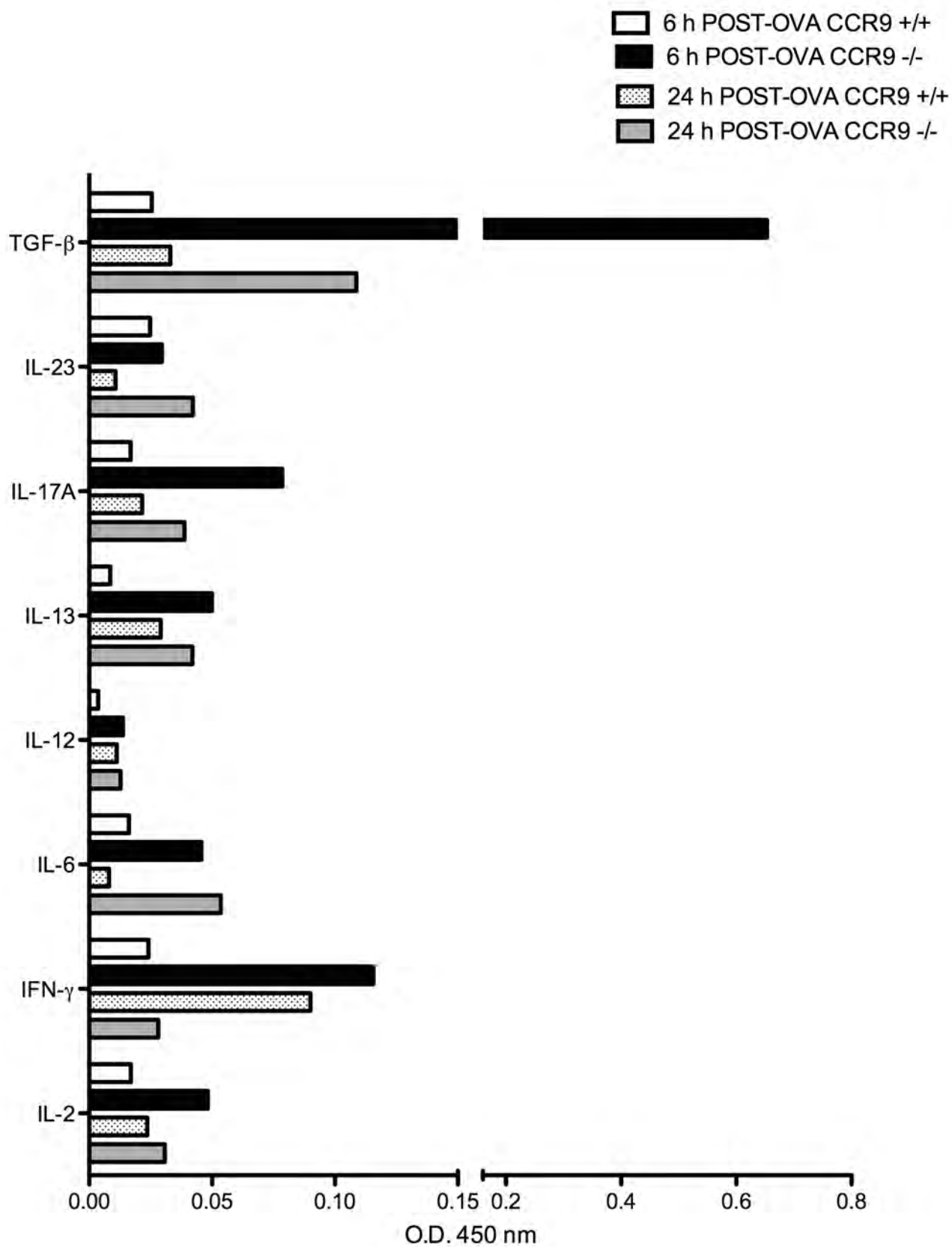

FIGURE S1

SS

6 h

24 h

Goat IgG  
Isotype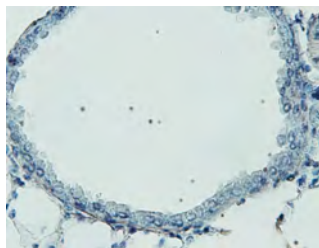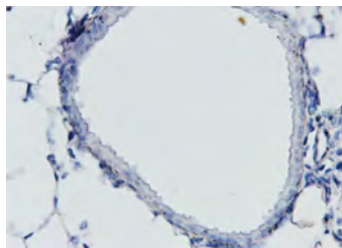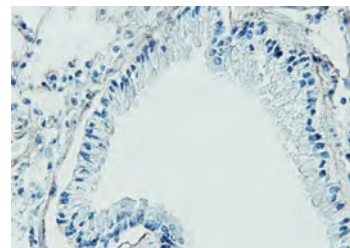

CCR9 +/+

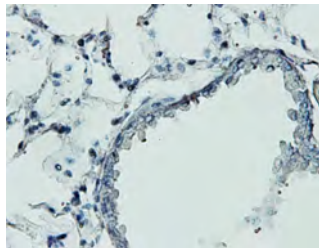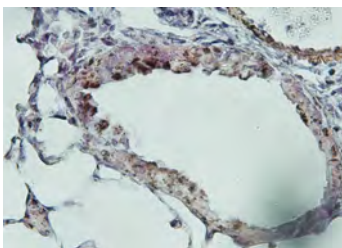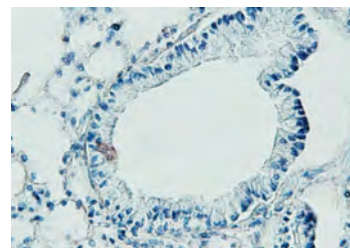

CCR9 -/-

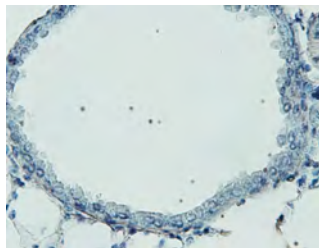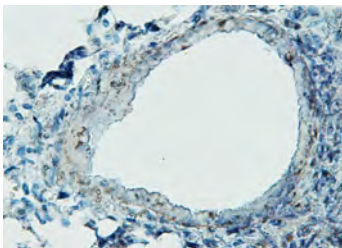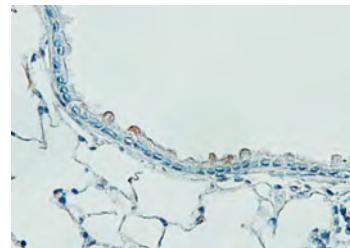

FIGURE S2

A

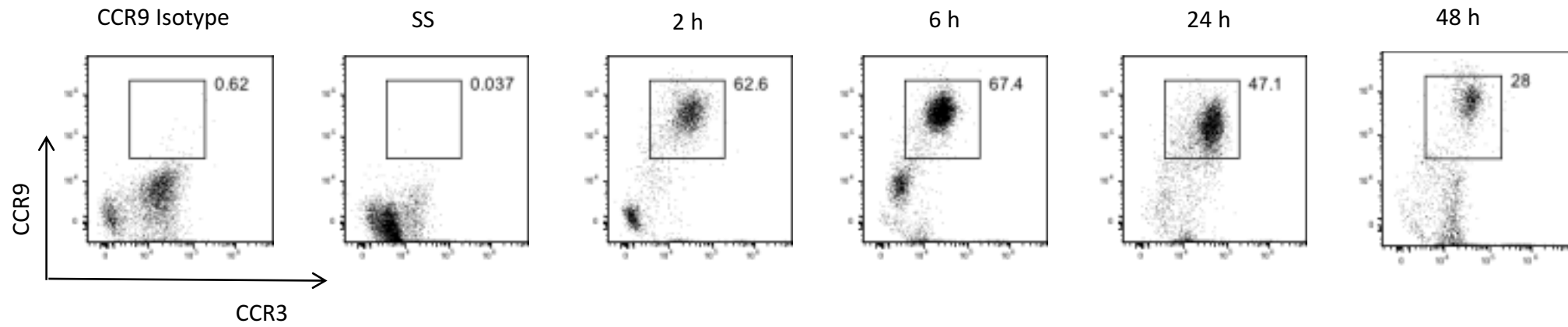

B

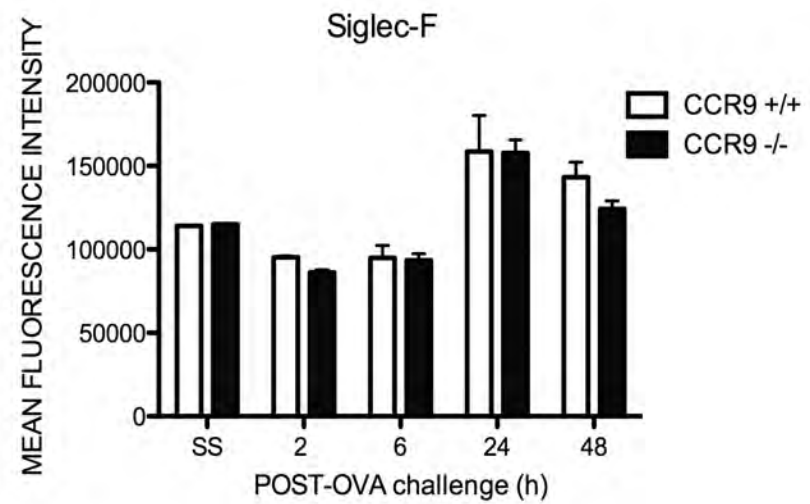

FIGURE S3

Supplement: Supplementary file 1 — Supplementary Figure 1. IL-2, IL-6, IL-12, IL-13, IL-17A, IL-23, IFN-g and TGF-b were determined from BAL by ELISA multiarray. CCR9 deficiency modifies the inflammatory cytokine production specially at 6 hours post OVA challenge. Supplementary Figure 2. CCL25 expression was analyzed in lung tissue by immunohistochemistry at 6 an 24 hours after challenge. Images demonstrate that CCL25 peaks is at 6 hours and is dependent on CCR9 expression. Supplementary Figure 3. Phenotypic analysis of eosinophils was performed by FACS in BAL-derived cells. CCR9 expression was determined and Siglec was not altered in the absence of CCR9. [file 3635809.f1.pdf]
